# Immunogenicity and efficacy of an oral live-attenuated vaccine for bovine Johne’s disease

**DOI:** 10.3389/fimmu.2023.1307621

**Published:** 2024-01-12

**Authors:** Razieh Eshraghisamani, Antonio Facciuolo, Victoria Harman-McKenna, Oscar Illanes, Jeroen De Buck

**Affiliations:** ^1^ Faculty of Veterinary Medicine, University of Calgary, Calgary, AB, Canada; ^2^ Vaccine and Infectious Disease Organization (VIDO), University of Saskatchewan, Saskatoon, SK, Canada; ^3^ Department of Veterinary Microbiology, Western College of Veterinary Medicine, University of Saskatchewan, Saskatoon, SK, Canada; ^4^ College of Veterinary Medicine, Long Island University, Brookville, NY, United States

**Keywords:** *mycobacterium avium* subsp. *paratuberculosis*, Johne’s disease, live-attenuated vaccines, modified live vaccine, oral vaccine, enteric/mucosal vaccine, dairy calves, immune responses

## Abstract

*Mycobacterium avium* subsp. *paratuberculosis* (MAP), the etiological agent of Johne’s disease (JD) in ruminants, establishes a prolonged and often lifelong enteric infection. The implementation of control measures for bovine JD has faced obstacles due to the considerable expenses involved in disease surveillance and hindered by unreliable and inadequate diagnostic tests, emphasizing the need for an effective vaccine that can stimulate mucosal immunity in the gastrointestinal tract. Previous investigations have demonstrated that deletion of the *BacA* gene in MAP produces an attenuated strain that can transiently colonize the calf small intestine while retaining its capacity to stimulate systemic immune responses similar to wildtype MAP strains. This study assessed the efficacy of the *BacA* gene deletion MAP strain, referred to as the *BacA* vaccine, when administered orally to young calves. The research aimed to evaluate its effectiveness in controlling MAP intestinal infection and to investigate the immune responses elicited by mucosal vaccination. The study represents the first evaluation of an enteric modified live MAP vaccine in the context of an oral MAP challenge in young calves. Oral immunization with *BacA* reduced MAP colonization specifically in the ileum and ileocecal valve. This partially protective immune response was associated with an increased frequency of CD4+ and CD8+ T cells with a pro-inflammatory phenotype (IFNγ+/TNFα+) in vaccinated animals. Moreover, re-stimulated PBMCs from vaccinated animals showed increased expression of *IFNγ*, *IP-10*, *IL-2*, and *IL-17* at 10- and 12-weeks post challenge. Furthermore, immunophenotyping of blood leukocytes revealed that vaccinated calves had increased levels of T cells expressing cell-surface markers consistent with long-term central memory. Overall, our findings provide new insights into the development and immunogenicity of a modified live MAP vaccine against bovine JD, demonstrating oral vaccination can stimulate host immune responses that can be protective against enteric MAP infection.

## 1 Introduction

*Mycobacterium avium* subsp. *paratuberculosis* (MAP) causes Johne’s disease (JD) in ruminants (1). Due to the limited efficacy of available killed vaccines (2, 3), control programs for JD have shifted from relying on vaccines to implementing management measures, such as separating infected and healthy animals, providing pasteurized milk for young stock, testing and culling infected animals, and educating producers about all risks of transmission (4, 5). The high prevalence of JD in livestock (6) combined with substantial costs for testing, treatment, and control measures, make it a significant financial burden for the dairy industry globally (7). In Australia and some European countries, a killed *Mycobacterium avium* vaccine called (Gudair) is used to effectively reduce the bacterial burden and delay the onset of clinical symptoms in sheep and goats (8). The killed *Mycobacterium avium* vaccine (Mycopar) for cattle has been discontinued in United States due to its limitations, including its inability to prevent the spread of the disease and cross-reactivity with bovine tuberculosis diagnostics (9–11). This underscores the need to develop a cost-effective protective vaccine that can prevent infection, block MAP transmission, and mitigate the negative production and economic outcomes associated with JD. Successful live-attenuated vaccines against other intracellular, enteric pathogens such as *Salmonella enterica* Serovar *Typhi (* 12) has sparked interest in developing a modified live vaccine for JD that can induce local cell mediated immunity. One such example is vaccination with a modified live MAP strain in which the *relA* gene was deleted induced an increase in effector T cells in the calf ileum cannulation model and limited the colonization of a virulent MAP strain (13). In another example, subcutaneous administration of a live *leuD* mutant of MAP resulted in reduced wildtype MAP colonization in goat intestinal tissue (14). Subcutaneous injection of a *pgsN* mutant also induced strong cell-mediated immune responses in blood, reduced bacterial burden in intestinal tissue and MAP fecal shedding in challenged goats and calves (15).

Similarly, there has been ongoing research in testing vaccines against JD in oral infection models. A live attenuated vaccine strain of MAP, ΔMAP1395c, was orally administrated in a caprine model. The study showed that the vaccine was safe and immunogenic but did not fully protect against infection with virulent MAP (16). The oral vaccine antigen delivery platform has shown some success in the Mycobacterium tuberculosis murine model when *Lactobacillus plantarums* expressing the MTB fusion antigen (Ag85B and ESAT-6) was used to vaccinate mice orally resulting in a reduction in bacterial burden (17). Whether genetically engineered strains of *Lactobacillus* are similarly immunogenic and efficacious in other animals, including ruminants, remains to be determined (18, 19).

The *BacA* gene is essential for the optimal growth of *Mycobacterium avium* subsp. *paratuberculosis* (MAP) in calf tissue (20, 21), and it is involved in the import of hydrophilic compounds such as vitamin B12 and iron metabolism. *Mycobacterium tuberculosis* mutant of *BacA* indicated diminished capacity to maintain a chronic infection (22). Deletion of this gene through specialized transduction produced a *BacA* live-attenuated strain, and a novel vaccine candidate, which our cohort confirmed was attenuated for colonization in the calf small intestine and induced immune responses capable of killing MAP *ex vivo*. The vaccine candidate also induced significantly greater inflammatory immune responses in peripheral immune cells in response to re-exposure to MAP antigens compared to the wildtype strain at early time points after infection (19).

The aim of the present study was to assess the effectiveness and immunogenicity of the oral *BacA* vaccine in preventing intestinal infection of young calves with MAP. This was accomplished by examining MAP colonization of the small intestine, monitoring MAP fecal shedding, and evaluating phenotypic and functional changes in the peripheral immune system. These combined approaches revealed new insights into the protective effects of the oral *BacA* vaccine against MAP infection and the corresponding immune responses elicited by enteric vaccination in calves.

## 2 Materials and methods

### 2.1 Bacterial strains and growth conditions

MAP A1-157 was cultivated in Difco Middlebrook 7H9 (Becton Dickinson and Company (BD), Sparks, MD, USA) media supplemented with 10% OADC (Oleic Albumin Dextrose Catalase; BD), 2 g/L mycobactin J (Allied Monitor Inc., Fayette, MO, USA) and 0.4% glycerol (Sigma, MO, USA). This strain belongs to a dominant clade that encompasses over 80% of all Canadian MAP isolates (23). *BacA*Δ, a deletion mutant of ABC transporter *BacA*, was grown in Difco Middlebrook 7H9 broth containing hygromycin (75 µg/mL). All bacterial cultures were incubated at 37°C with shaking at 225 rpm and the bacterial inoculum dose was quantified using the wet weight method.

### 2.2 Calf trial

**Figure 1:**
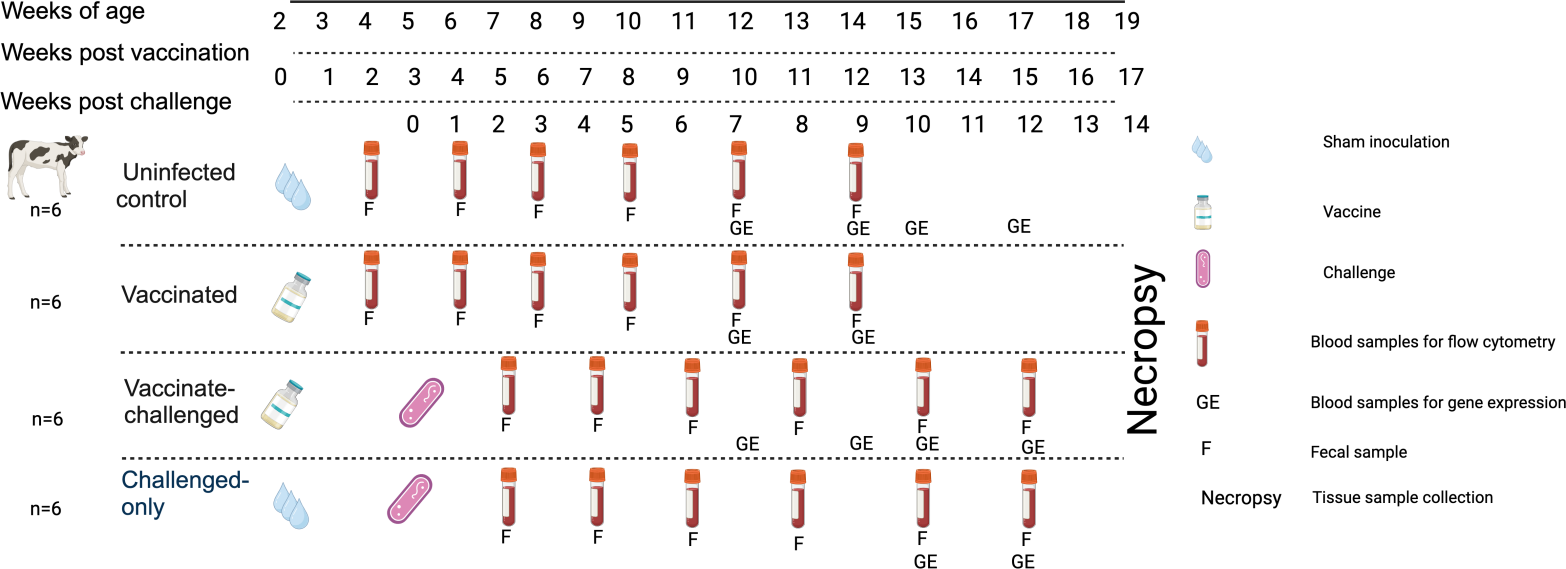
Experimental Design and Sampling Schedule. Uninfected control group underwent sham inoculation at 2 weeks of age. Fecal samples were collected from uninfected control at 2, 4, 6, 8, 10 and 12-weeks post sham inoculation. Blood samples for flow cytometric analysis were collected from uninfected control group at 2, 4, 6, 8, 10 and 12-weeks post sham inoculation. Blood samples for gene expression analysis were collected from uninfected control group at 10, 12, 13 and 15-weeks post sham inoculation. The Vaccinated group received the oral vaccine at 2 weeks of age. Fecal samples were collected from vaccinated group at 2, 4, 6, 8, 10 and 12-weeks post vaccination. Blood samples for flow cytometric analysis were collected from vaccinated group at 2, 4, 6, 8, 10, and 12-weeks post vaccination. Blood samples for gene expression analysis were collected from vaccinated group at 10 and 12-weeks post vaccination. The vaccinated-challenged group received the oral vaccine at 2 weeks of age and received the challenge at 5 weeks of age. Fecal samples were collected from vaccinated-challenged group at 2, 4, 6, 8, 10 and 12-weeks post challenge. Blood samples for flow cytometric analysis were collected from vaccinated-challenged group at 2, 4, 6, 8, 10 and 12-weeks post challenge. Blood samples for gene expression analysis were collected from vaccinated-challenged group at 10 and 12-weeks post challenge. The challenged-only group received the challenge at 5 weeks of age. Fecal samples were collected from challenged-only group at 2, 4, 6, 8, 10, and 12-weeks post challenge. Blood samples for flow cytometric analysis were collected from challenged-only group at 2, 4, 6, 8, 10 and 12-weeks post challenge. Blood samples for gene expression analysis were collected from challenged-only group at 10 and 12-weeks post challenge. Animals were all sacrificed at 18 weeks of age and tissue samples were collected.

Upon approval by the Veterinary Sciences Animal Care Committee (VSACC) of the University of Calgary, animal care protocol AC21-0199 was implemented and all procedures were carried out in accordance with the Standard Operating Procedures (SOPs) specified therein. Twenty-four Holstein-Friesian bull calves were procured from dairy farms in Alberta with a low occurrence of JD, and they were all acquired within 12 hours of their birth. Individual housing units, tailored to specific enrichment requirements, were established within a biosecurity level 2 housing facility at the Veterinary Science Research Station (VSRS). A total of 24 calves were divided into four cohorts through an opportunistic randomization process. The first cohort consisted of 6 non-infected healthy controls, the second cohort included 6 calves that were vaccinated with *BacA*Δ, the third cohort included 6 calves that were first vaccinated with *BacA* and then challenged with virulent MAP, and the fourth cohort included 6 calves that were challenged with virulent MAP. The cohorts that received the vaccination were given an oral inoculation of 10^9^ colony-forming units (CFU) of the *BacA* in two doses over the course of two consecutive days when they were two weeks old. Infected cohorts were orally challenged with 2×10^9^ CFU of MAP strains in two consecutive days at five weeks of age (**Figure 1**).

### 2.3 Tissue samples

#### 2.3.1 Tissue sample collection and homogenization

The calves were sacrificed at 4.5 months (20 weeks) old through an intravenous injection of barbiturate (Sodium pentobarbital; trade name Euthanyl Forte®, DIN 00241326, Bimeda-MTC Animal Health Inc., ON, Canada) after being sedated with xylazine (0.3 mg/kg). Whole tissue samples were collected from six intestinal sites: the ileocaecal valve, the terminal small intestine containing visible continuous Peyer’s patch and extending into the ileocecal fold, the mid-jejunum, and a single draining mesenteric lymph node (MLN) belonging to each of the afore mentioned anatomical sites. To avoid cross-contamination, sanitized tools and fresh gloves were employed for every tissue sample, while zip ties were utilized to demarcate and isolate sample areas prior to collection, thus preventing any shifting of intestinal contents. The whole tissue samples were homogenized for culture and DNA extraction.

Samples were taken to a BSL-2 laboratory and processed within 4 hours of collection. The whole tissue was homogenized as previously described (24). The mucosa layers of the ileum, distal jejunum, and ileocaecal valve were scraped using microscope slides, with a new slide used for each sample. The fatty tissue was removed from the MLNs before they were fragmented into smaller pieces. To homogenize a combined 2.5 g of the samples, GentleMACS M tubes (Miltenyi Biotech Inc., Auburn, CA, USA) were utilized with 10 mL of PBS solution containing 0.5% triton X-100 (Sigma). For each sample, the dissociation process was carried out for 53 seconds with 2753 rounds per run (rpr) and repeated three times. Two homogenized samples were prepared for each sample, with one used for tissue culture and the other for direct DNA extraction.

#### 2.3.2 MAP bacteria recovery from tissues

The homogenized samples were transferred to a 50 mL falcon tube and centrifuged at 4700 × g for 20 min. The resulting pellet was suspended in 25 mL of 0.75% Hexadecyl pyridinium chloride (HPC, Sigma) and half-strength Brain Heart Infusion (BHI) broth (Sigma), along with 10 sterile 4-mm glass beads (Sigma), and vigorously vortexed for 2-3 min. The samples were then incubated at 37°C for 3 h, followed by a 15 min centrifugation at 4700 × g. After resuspending the pellet in 3 mL of antibiotic brew (consisting of 0.2 mL paraJEM® AS, 1.5 mL full-strength BHI, and 1.3 mL ddH2O) (TREK Diagnostic Systems, Cleveland, OH, USA), the samples were incubated overnight at 37°C. Subsequently, 1 mL of the sample was cultured in ParaJEM culture bottles for 49 days.

#### 2.3.3 DNA extraction from homogenized tissue

Enzymatic digestion was employed to extract and detect MAP DNA from intestinal tissue and lymph nodes (25). To pellet the debris, the homogenized samples in 10 mL PBS were centrifuged at 200 × *g* for 5 min after a short vortex. Supernatants were collected in new 50-mL conial tubes which were then centrifuged at 5200 × *g* for 20 min to pellet MAP cells. Pellets were resuspended in 2.5 mL TE buffer (10 mM tris-chloride, 1 mM EDTA, pH 8.0) and aliquoted to 2-mL screw cap tubes with ZR Bashing Beads (Zymo Research, USA). Samples were centrifuged at 500 × *g* for 5 min after 2 min of bead beating. The supernatants were transferred into sterile 2-mL microcentrifuge tubes containing lysozyme (5 mg/mL; Sigma) and incubated at 37°C for 2 h. After 2 h, samples were supplemented with SDS (1 mg/mL) and proteinase k (2 mg/mL; Sigma) and further incubated at 56°C overnight (16-20 hrs). Following the proteinase k digest, 0.4 volume of 5 M potassium acetate (Thermofisher scientific) was slowly added to each sample under gentle mixing and incubated for 10 min on ice before. centrifuging at 9600 × g for 12 min. To purify the DNA, an equal volume of tris-saturated phenol:chloroform:isoamyl alcohol (25:24:1) (Sigma) was added to the supernatant and the tubes were inverted 20 times before being centrifuged at 9600 × g for 12 min. The resulting aqueous phase was then isolated by centrifugation at 9600 × g for 12 min. To precipitate the DNA, 1/10 volume of cold 3 M sodium acetate (Sigma) and 2.5 volumes of absolute ethanol were added to the aqueous phase, and the mixture was incubated at -80°C for 2 h. The DNA was then precipitated by centrifugation at 15000 rpm for 20 min, washed with 70% ethanol, and resuspended in 50 μLTE buffer. The DNA clean and concentrator kit (Zymo Research) was employed to remove the potential PCR inhibitors.

#### 2.3.4 Histology

Tissue samples were collected for histology from the continuous band of Peyer’s patch in the ileum and discrete Peyer’s patches in the jejunum in addition to their MLNs. Samples were placed in a labeled cassette, immersed in 10% neutral buffered formalin, routinely processed for histological assessment, embedded in paraffin and sectioned and stained with Hematoxylin-Eosin (HE) as well as Ziehl-Neelsen for the detection of acid-fast positive bacteria. Slides were examined by light microscopy and scored for paratuberculosis-associated histological lesions using the scoring system proposed by González et al. (26). This scoring system ranges from 0 (no lesions) to 3 (diffuse lymphocytic, multibacillary lesions), with intermediate scores indicating focal (1) or multifocal lesions (2). Microscopic assessment of the slides was performed by an experienced veterinary pathologist unaware of the inoculation status of the calves.

### 2.4 Fecal samples

#### 2.4.1 Fecal sample collection and culture

Fecal samples were obtained at various time points (12 h, 2, 4, 6, 8, 10, 12, 14 weeks) after inoculation of the oral vaccine in vaccinated cohort and after challenge with virulent MAP in vaccinated-challenged and challenged-only cohort. Fecal samples were processed using TREK para-JEM culture media (TREK Diagnostic Systems), following previously described methods (6). Briefly, 2 g of feces was mixed with 30 mL of autoclaved distilled water in a 50 mL conical tube and incubated at room temperature for 30 min. Next, 5 mL of the supernatant was transferred to a solution of 0.9% HPC and half-strength BHI and incubated for 24 h at 37°C for decontamination. The samples were then centrifuged, and the pellet was resuspended in a mixture of antibiotic solution provided by the kit, water, and full-strength BHI, followed by another 24-hour incubation at 37°C. Finally, 1 mL of the supernatant was added to liquid culture medium in TREK para-JEM culture bottles and incubated at 37°C for 49 days.

#### 2.4.2 DNA extraction from fecal samples

The MagMax DNA Multi-sample kit (ThermoFisher Scientific) was utilized as per the manufacturer’s guidelines to extract DNA directly from fecal samples. In brief, 0.3 ± 0.1 g of the fecal sample was mixed with 1 mL PBS in a thermomixer at 1300 × g for 3 min. The resulting supernatant was combined with 235 μL binding solution in pre-filled tubes containing 0.1 mm zirconium beads. The lysate was subjected to bead beating twice for 5 min, with a 5 min pause in between. The lysate was transferred to a 96-well plate after centrifugation at 16,000 × g for 3 min. Isopropanol was added to the wells, and the plate was shaken at 500 rpm for 1 min, followed by the addition of magnetic beads, which were shaken at 550 rpm for 5 min. The supernatant was collected and discarded using a magnetic stand. The beads were rinsed twice with wash solution 1 and 2, and the supernatant was discarded after each wash step. Following the second wash, the plate was shaken at 550 rpm without the lid for 3 min to dry the beads. The elution buffer was added and shaken at 550 rpm for 3 min, and the DNA-containing elution buffer was obtained using the magnetic stand. The purified DNA was preserved at -20°C until needed.

### 2.5 DNA extraction from tissue and fecal cultures

After the 49-day incubation period, the identical method was applied to extract DNA from the tissue and fecal cultures. A mixture of 800 mL of 100% ethanol and 200 mL of the culture broth was created, which was then centrifuged for 9 minutes at 7500 rpm. The resultant pellet was washed twice with Dulbecco’s Phosphate Buffered Saline (DPBS; Gibco, Carlsbad, CA, USA) and boiled in 100 mL of sterile UltraPure DNase/RNase-Free Distilled Water (Gibco) at 100°C for 30 min. The supernatant was then transferred to a new, sterile 1.5 mL snap-cap microcentrifuge tube after a final centrifugation stage at 7500 rpm for 2.5 min. The DNA clean and concentrator kit (Zymo Research) was employed to remove the potential PCR inhibitors.

### 2.6 MAP detection qPCR

#### 2.6.1 qPCR for detection and quantification of MAP DNA from tissue and fecal samples

The two-duplex real-time qPCR system was utilized to detect MAP DNA by amplifying MAP-specific DNA elements *IS900* and *F57 (* 27). An internal amplification control (IAC) plasmid containing *IS900* or *F57*, is amplified using the same primers alongside the test samples. The 20 μl reaction mixture for both target sequences contained the following components: 10 μl of TaqMan Fast Advanced Master Mix (Invitrogen), 10 pmol of primers (Integrated DNA Technologies (IDT), CA, USA), 1 pmol of FAM-ZEN-IOWABLACKQ target probe (IDT), 1 pmol of CY5-TAO-IOWABLACKQ IAC probe (IDT), 50 copies of IAC plasmid, and 50 ng of DNA template. The qPCRs were conducted using a Bio-Rad CFX96 thermocycler at the following conditions: 50°C for 2 min, then proceed with a denaturation at 95°C for 20 min, and finally conduct 40 cycles of annealing for 3 s at 95°C and extension for 30 s at 60°C. A plasmid harboring the *F57* genewas used to construct a standard curve to quantity MAP copies per gram of tissue.

### 2.7 Flow cytometric analysis of peripheral blood mononuclear cells

#### 2.7.1 Panel design and gating strategy

**Figure 2:**
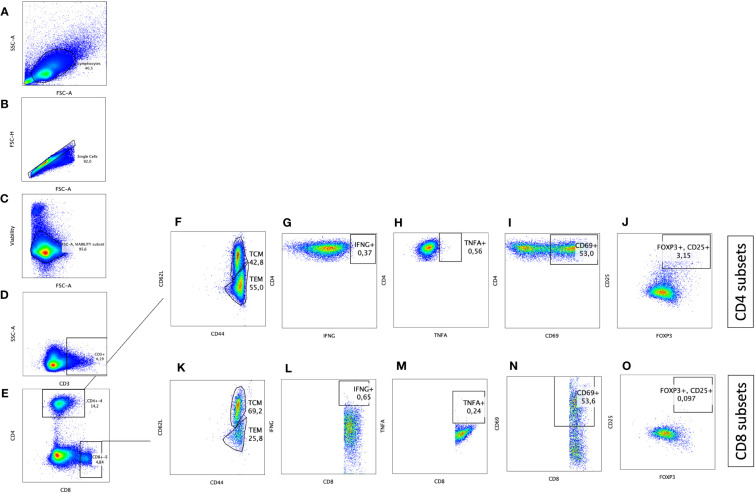
Immunophenotyping of CD4+ T helper and CD8+ cytotoxic T cells with pro-inflammatory, anti-inflammatory, memory, and activation phenotypes. Gating strategy selected for lymphocytes based on size **(A)**, cell singlets **(B)**, exclusion of non-viable cells **(C)**, and finally on CD3 staining for T cell identification **(D)**. T helper cells were differentiated from cytotoxic T cells based on CD4 and CD8 expression, respectively **(E)**. CD4+CD3+ and CD8+CD3+ subsets were further defined by plotting on **(F, K)** CD44 and CD62L to differentiate T effector memory (CD62L-CD44+)versus T central memory (CD62L+CD44+), on **(G, L)** IFNG to identify IFNG producing cells, on **(H, M)** TNFA to identify TNFA producing cells, on **(I, N)** CD69 to identify activated T cells, and on **(J, O)** CD25 and FOXP3 to define T regulatory cells (CD25+FOXP3+).

Blood samples were obtained at 2, 4, 6, 8, 10 and 12 weeks after inoculation of the oral vaccine in vaccinated cohort and after challenge with virulent MAP in vaccinated-challenged and challenged-only cohort. Peripheral blood mononuclear cells (PBMCs) were isolated using previously described methods (28). Flow cytometry analysis was conducted by incorporating direct surface and intracellular staining methods. A twelve-color panel was designed to detect T helper cells (CD3+CD4+) and cytotoxic T cells (CD3+CD8+) with various phenotypes, including central memory (CD62L+CD44+), effector memory (CD62L-, CD44+), IFNγ producing (IFNγ +), TNFα producing (TNFα+), regulatory (CD25+FOXP3+), and acute activated (CD69+). The stepwise gating strategy is shown in **Figure 2**.

#### 2.7.2 Conjugated antibody staining for surface and intracellular markers

A total of 2×10^6^ cells per sample were transferred into the wells of 96 well round-bottom plates (Greiner Bio-One, NC, USA) and centrifuged at 400 × g for 5 min. The cells were washed with PBS and pellet by centrifugation at 400 ×g for 5 min. The resulting pellet was resuspended in 50 μl of diluted eF-506 viability dye (Invitrogen™ eBioscience™) and incubated for 20 minutes at 4°C. After a wash with FACS buffer (Invitrogen™ eBioscience™), cells were labelled with a cocktail of ten fluorochrome-conjugated mouse antibodies specific for bovine CD antigens: CD3-APCCY5(Novus Biologicals, CO, USA), CD4-PASIFICBLUE (Bio-Rad), CD8-DYLIGHT594 (Novus Biologicals), CD62L-BV650 (Biolegend, CA, USA), CD44-PECY5 (Biolegend), -CXCR3-BV771 (Biolegend), LAG3-BUV661 (BD Bioscience, USA), CD25-PE (Invitrogen), and CD69-ALEXAFLOUR647 (Bio-Rad). Cells were incubated for 30 min on ice. After a single wash, cells were incubated with fixation/permeabilization buffer (Invitrogen) for 20 minutes at 4°C to fix the surface staining and permeabilize the cell wall for intracellular staining. The permeabilized cells were then labelled with a mixture of three fluorochrome-conjugated mouse antibodies specific for bovine IFNγ-PERCP (Novus Biologicals), TNFα-BV605 (US Biologicals, USA), and FOXP3-PECY7 (Invitrogen) diluted in permeabilization solution. After a final wash, cells were resuspended in 150 μl of FACS buffer for acquisition with Cytek Bioscience flow cytometer (Cytek Bioscience, California, USA). For every fluorochrome used, both a single-color control and a fluorescent minus one (FMO) control were incorporated. Additionally, an unstained control was included for each sample type in all panels. The unstained and FMO controls were used to set the gates for each marker.

### 2.8 Gene expression assay

#### 2.8.1 Preparation of MAP whole cell lysate

A log-phase culture of MAP A1-157 was grown in Difco Middlebrook 7H9 Broth supplemented with 10% Middlebrook OADC enrichment (BD) and 2 mg/L ferric mycobactin J (Allied Monitor Inc.). The MAP cells were centrifuged at 12,000 × g for 5 minutes, and then resuspended in lysis buffer (consisting of PBS, 5% glycerol, 5 mM EDTA, 1 mM PMSF) and homogenized with 0.1 mm Zirconia/Silica beads using a Mini Bead Beater-96 (Biospec, OK, USA) in 5 cycles of 25 seconds each (29). The protein concentration of the resulting clarified supernatant was determined using a Pierce BCA Protein Assay Kit (Thermo Fisher Scientific, Inc.). The resulting lysate were stored at a temperature of -20°C in separate aliquots.

#### 2.8.2 *Ex vivo* stimulation assay

Blood samples were obtained 10 and 12 weeks after vaccination from vaccinated cohort and 10 and 12 weeks after challenge from challenged cohorts. PBMCs were cultured in 12-well plates with a total of 2 million cells per well and incubated with either medium alone or 5 μg/mL MAP whole cell lysate. The cells were incubated for 24 h at 37°C, 5% CO_2_ in a humidified incubator, and then centrifuged at 300 × g for 7 minutes. The supernatant was discarded, and the remaining cell pellet was lysed with 1 mL of TRIzol™ (Invitrogen) per well. The lysed samples were stored at -80°C until RNA extraction.

#### 2.8.3 RNA extraction

The stored samples in 1 mL of TRIzol were thawed to room temperature. To extract RNA, 0.2 mL of chloroform (ThermoScientific Chemicals) was added to each tube, and the tubes were vigorously mixed by inverting 20 times and incubated at room temperature for 5 minutes. The samples were then centrifuged at 12000 × g for 15 min, and the aqueous phase was transferred to new tubes, being careful not to disturb the protein-containing interphase layer. RNA was purified from the aqueous phase using a RNeasy kit following the manufacturer instructions and stored at -80C. The assessment of RNA integrity, quality, and quantity was conducted utilizing an Agilent 2100 BioAnalyzer and a Nanodrop™ Spectrophotometer (ThermoFisher Scientific).

#### 2.8.4 Reverse transcription

The QuantiTect Reverse Transcription kit (Qiagen) was used following the manufacturer instructions. To ensure that the amount of cDNA was consistent across all samples, 400 ng of RNA from each sample was reverse transcribed to cDNA. The resulting cDNA was stored at -20°C.

#### 2.8.5 Gene expression analysis

Real-time qRT-PCR was used to measure gene expression for several pro-inflammatory cytokines, such as *IFNγ*, *TNFα*, *CXCL10*, *IL-2*, *IL-15*, *IL-12*, *IL-18*, *IL-27*, and *IL-17*, anti-inflammatory cytokines, such as *TGFβ*, *IL-4*, and *IL-10*, and T cell transcription factors, including *T-bet* and *FOXP3* (**Supplementary Table 1**). The Applied Biosystems™ PowerTrackTM SYBR Green Master Mix was used according to the manufacturer’s instructions to prepare a 10 μl reaction mixture containing SYBR Green Master Mix, forward and reverse primers, and Yellow Sample Buffer with 10 ng of cDNA template. The qRT-PCR conditions involved initial denaturation at 95°C for 15 s followed by 40 cycles of 1 min at 60°C and 15 s at 95°C. The amplification reaction was then followed by a melting step to check the specificity of the amplicons. The mRNA levels of genes of interest were normalized using *H3F3A* gene as an endogenous expression control (**Supplementary Table 1**). The level of gene transcription in PBMCs after stimulation with 5 μg/mL MAP whole cell lysate was compared to non-stimulated cells as a calibrator, with upregulation and downregulation of genes in stimulated cells compared to the non-stimulated homolog of the same sample. The 2^-ΔΔct^ method was used to analyze the data. The results were presented as relative gene transcription compared to the unstimulated control.

### 2.9 Data analysis

All statistical analysis were conducted using prism software *ver*. 9.2.0 (GraphPad Software, San Diego, California). The difference in the numbers of animals that are positive for MAP culture or tissue between cohorts were analyzed by chi-square and Fisher tests. The results of relative abundance of immune cells in flow cytometry were analyzed using two sample t-test. The results of gene expression assay and were analyzed using one-way ANOVA with Bonferroni correction. The comparison of means between cohorts were performed employing Tukey-Kramer *post hoc* test. P-values less than 0.05 were deemed statistically significant.

## 3 Results

### 3.1 Oral *BacA* vaccine can control intestinal MAP infection in ileum and ileocecal valve

To determine the efficacy of the oral *BacA* vaccine we quantified MAP tissue burden in the ileum, jejunum, ileocecal valve, and draining MLNs for all calves in the vaccinated-challenged cohort and compared this to the challenged alone cohort. DNA was extracted from these tissues directly to test for MAP specific DNA. DNA extraction was additionally conducted on bacterial cultures obtained after 49 days, facilitating the cultivation and amplification of viable MAP from tissue to enhance the sensitivity of detection. The extracted DNA was then subjected to qPCR targeting the MAP-specific single copy DNA element *F57*. Animals were deemed positive if MAP DNA was directly detected in tissue samples or MAP DNA was detected in para-JEM broth cultures.

**Figure 3:**
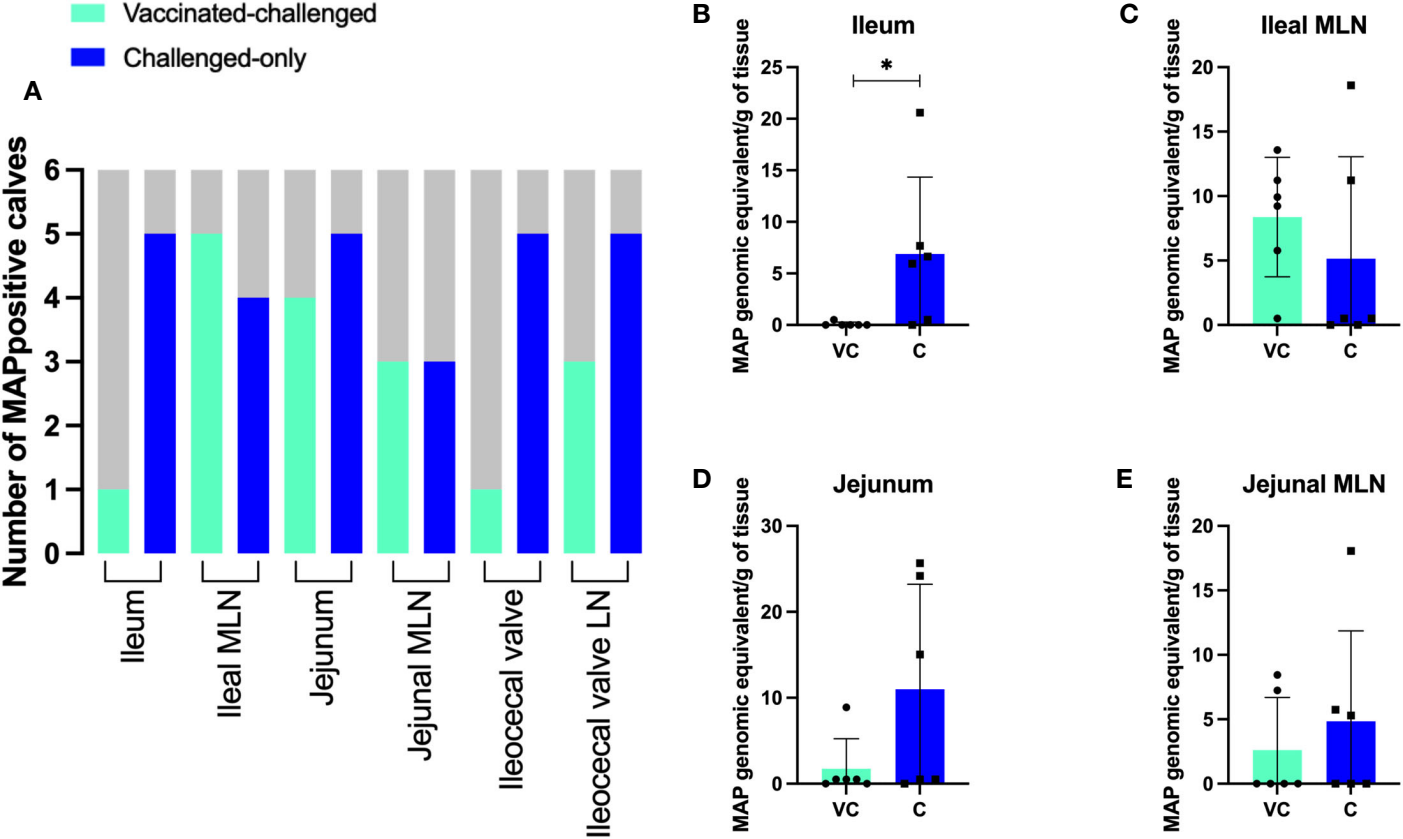
**(A)** The number of MAP positive animals in each cohort. DNA was extracted directly from homogenized tissue. Additionally, the homogenized tissue was cultured using the ParaJEM culture system. After an incubation period of forty-nine days, DNA was extracted from the culture broth. The presence or absence of MAP DNA was assessed in 50 ng of template using F57 qPCR. The variation between cohorts was analyzed using a Chi-square-test. **(B–E)** MAP burden in intestinal tissue and MLNs. To quantify the genomic equivalents (GE) of MAP in 1 gram of tissue, F57 qPCR was utilized. The variation between cohorts was analyzed using a two-sample t-test. (VC, vaccinated-challenged; C, challenged). *: p<0.05.

In the challenge-only cohort, five animals were positive for MAP in ileum, jejunum, ileocecal valve, and ileocecal valve LN. In the same cohort, MAP was detected in the ileal MLN of four animals and in the jejunal MLN of three animals (**Figure 3A**). However, in the vaccinated-challenge cohort, MAP was only detected in the ileum and ileocecal valve of one animal. Within the vaccinated-challenged cohort, five animals were positive for MAP in the ileal MLN, and four animals were positive in the jejunum. In the same cohort, three animals were positive for MAP in both the jejunal MLN and the ileocecal valve LN (**Figure 3A**). MAP was not detected in any of the animals belonging to the uninfected control cohort. Similarly, MAP was also not detected in any of the animals belonging to the vaccinated cohort consistent with our previous observations that *BacA* deletion in MAP attenuates persistent infection in calves.

Quantification of MAP using the single copy DNA element *F57* revealed significantly greater bacterial burden in the ileum of animals in the challenged-only cohort when compared to those in the vaccinated-challenged cohort (**Figure 3B**). The average of MAP genomic equivalent recovered from a gram of ileum was <1 and 6.9 in vaccinated-challenge and challenged-only cohorts, respectively. There was no difference (P > 0.05) in mean MAP recovery when comparing the ileal MLN, jejunum, and jejunal MLN between vaccinated-challenged to challenged-only animals. (**Figures 3C–E**). Due to the small size of the ileocecal valve, samples collected from this tissue were exclusively used for culturing rather than direct DNA extraction, as the preference for culture was based on its higher sensitivity.

**Figure 4:**
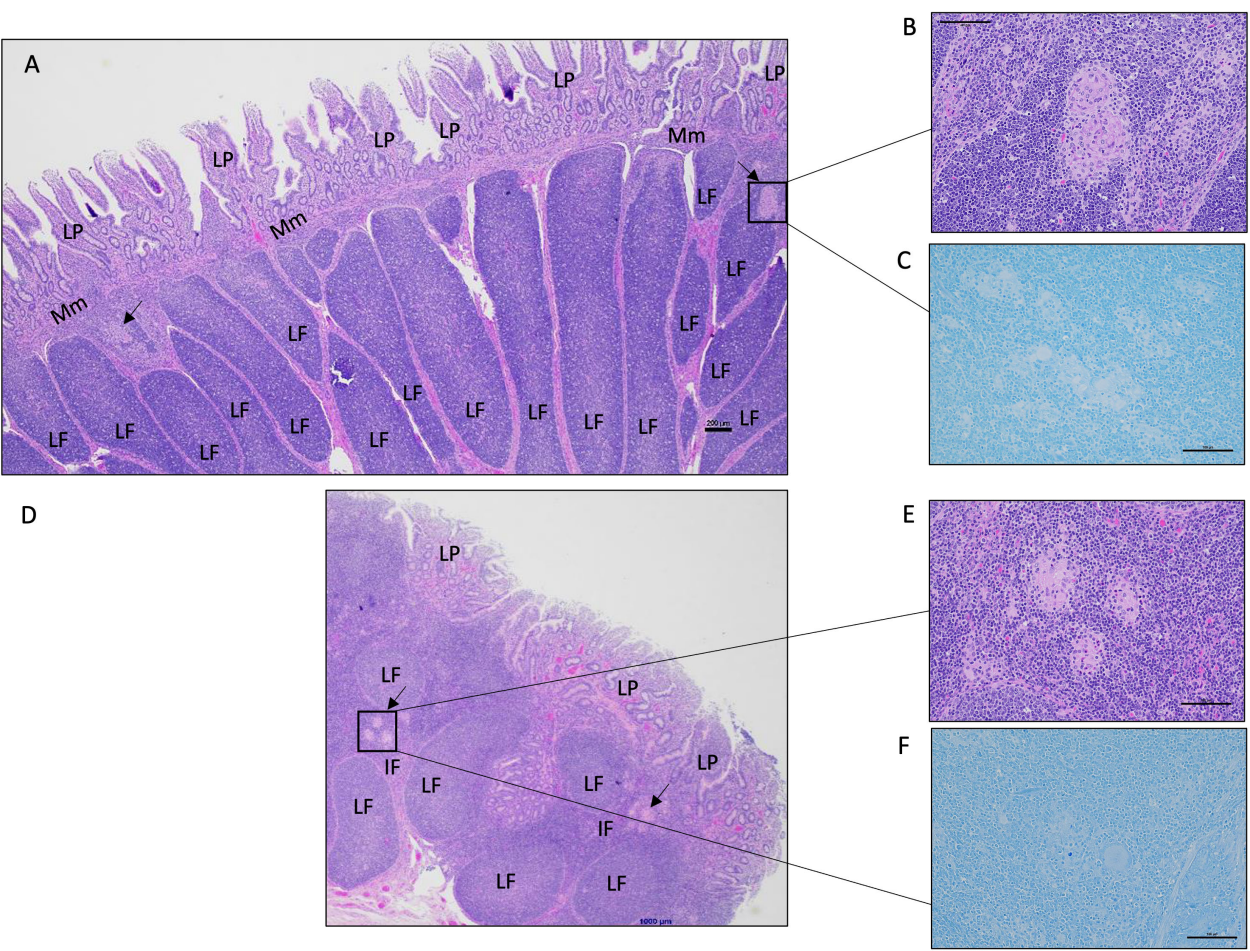
Representative histology images of the calf small intestine following MAP challenge. **(A)** Image of a continuous Peyer’s patch in the ileum from a vaccinated-challenged calf. Boxed image and arrows highlight focal lesions in the interfollicular regions in the submucosa (200 μm, HE). **(B)** A magnified image of the granulomatous inflammation observed in **(A)** (100 μm, HE). **(C)** In the same vaccinated-challenged calf, there was no detectable acid-fast stained organisms (100 μm, acid-fast stain). **(D)** Image of a discrete Peyer’s patch in the mid-jejunum from a challenged-only calf with a focal area of granulomatous inflammation within the interfollicular region indicated by the arrow and boxed region (200 μm, HE). **(E)** A magnified image of the granulomatous inflammation observed in **(D)** (100 μm, HE). **(F)** In the same challenged-only calf, acid-fasted organisms were not detected (100 μm, acid-fast stain). LP, lamina propria; Mm, muscularis mucosae; LF, lymphoid follicle; IF, interfollicular region.

Histological analysis of intestinal tissue samples from uninfected control and vaccinated calves did not reveal any visible lesions, and staining for acid-fast bacteria was negative in all tissue samples. Only in two of the six animals in the vaccinated-challenged cohort, small foci of granulomatous inflammation confined. These inflammatory regions were mild in severity and primarily localized within the submucosa and Peyer’s patches of the small intestine (**Figure 4A**). In, five of the six animals in the challenged-only cohort, small foci of granulomatous inflammation in the submucosa of small intestine were identified (**Figure 4D**). Of these, two cases were classified as mild, and three showed a multifocal pattern of inflammation. No acid-fast organisms were detected in any of these calves (**Figures 4C, F**). Only one animal belonging to the challenged-only cohort had a visible lesion in the ileal MLN.

### 3.2 Oral *BacA* vaccine can reduce MAP fecal shedding

To assess the efficacy of the oral *BacA* vaccine on MAP shedding in feces, samples were collected every two weeks after challenge. DNA was extracted from fecal cultures and directly from fecal samples of animals that were positive for MAP DNA in the cultures. The extracted DNA was then subjected to qPCR targeting the MAP-specific *F57* and *IS900* DNA elements. None of the animals in the uninfected control cohort were positive for MAP in fecal samples. All animals that were challenged with MAP had detectable levels of MAP in their feces 24 hours after challenge likely representing passive shedding.

Two weeks after the challenge, fecal samples from two animals in the challenge-only cohort were positive for MAP. At four-weeks and six-weeks post challenge four animals in challenge-only cohort were MAP positive, and two animals were positive at the eight-week post challenge. Starting from week 10 onward, only one animal had detectable levels of MAP (**Table 1**).

**Table 1:** MAP fecal shedding.

	Weeks post vaccination and/or challenge						
Cohort	2	4	6	8	10	12	14
Uninfected Control	–	–	–	–	–	–	–
Vaccinated	5	3	4	–	–	–	–
Vaccinated-challenged	3	5	4	–	–	–	–
Challenged-only	2	4	4	2	1	1	1

Fecal samples were cultured using the ParaJEM culture system. After an incubation period of forty-nine days, DNA was extracted from the culture broth and qPCR used to identify F57 and IS900 MAP-specific DNA elements. Uninfected controls received neither oral challenge with MAP nor the oral BacA vaccine. Vaccinated cohorts received the oral BacA modified live vaccine. Vaccinated-challenged cohort received the BacA modified live vaccine at 2 weeks and were challenged with a virulent MAP strain at 5 weeks. Challenged cohort received only the virulent MAP strain. The number in each cell represents the overall count of animals, out of a total of six in that cohort, in which MAP was detected in feces during the specified time interval.

Two weeks after challenge, fecal samples from three animals in the vaccinated-challenged cohort were positive for MAP. At four weeks after challenge, five animals in the vaccinated-challenged cohort were MAP-positive. Six weeks after challenge, fecal samples from four animals in the same cohort were MAP-positive. Starting from week 8 onward, none of the animals within the same cohort showed positive results (**Table 1**).

Two week following vaccination, fecal samples from five animals in the vaccinated cohort were positive for MAP. At four weeks post vaccination, three animals in the vaccinated cohort were MAP positive, and four animals were positive at the six-week post-vaccination. Starting from week 8 onward, MAP was not detectable in any of the vaccinated animals. At all timepoints, MAP was undetectable in the uninfected control calves (**Table 1**).

The vaccine strain of MAP contains a hygromycin resistance cassette. By targeting and detecting the presence of both the hygromycin resistance gene and *F57*, the vaccine strain could be differentiated from the virulent strain in samples that were MAP-positive. Some of the positive samples from the vaccinated cohort showed hygromycin presence with Ct values very close to the cut-off point of 37. None of the MAP positive samples from vaccinated-challenged and challenged-only cohorts were positive for hygromycin resistance gene.

### 3.3 Oral *BacA* vaccine induces CD4+ and CD8+ T cells with pro-inflammatory phenotypes

IFNγ and TNFα-producing CD4+ and CD8+ T cells have been implicated in providing a protective immune response against intracellular mycobacterial infections. Flow cytometry analysis was used to measure the frequency of IFNγ- and TNFα-producing CD4+ and CD8+ T cells in peripheral blood mononuclear cells (PBMCs) at 2, 4, 6, 8, 10, and 12 weeks after inoculation of the oral vaccine for the vaccinated cohort and after challenge with virulent MAP in vaccinated-challenged and challenged-only cohort.

**Figure 5:**
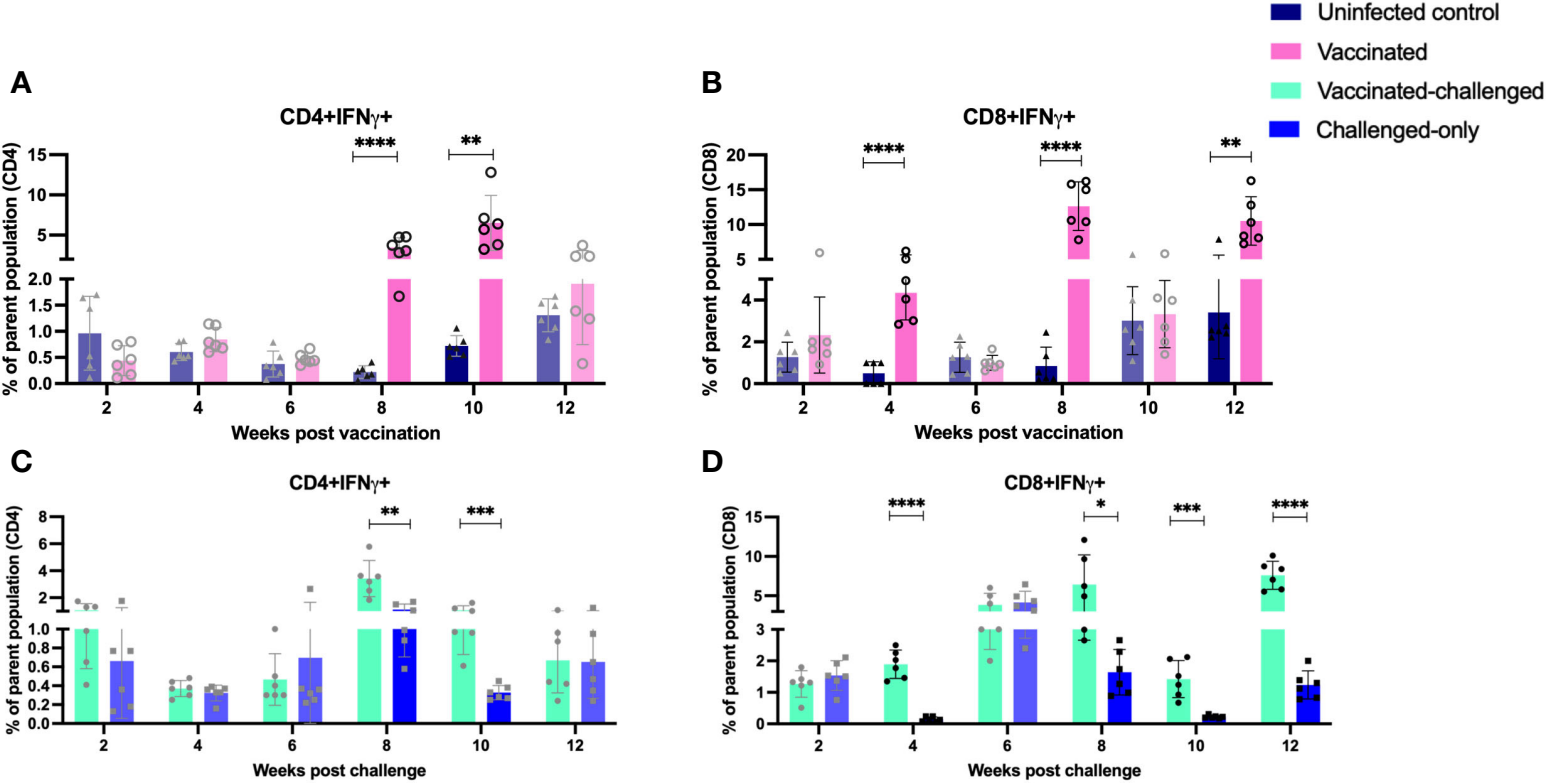
Immunophenotyping of IFN-gamma producing T cells. PBMCs were isolated from vaccinated and uninfected control cohorts at 2, 4, 6, 8, 10, 12 weeks post vaccination. PBMCs were isolated from vaccinated-challenged and challenged-only cohorts at 2, 4, 6, 8, 10, 12 weeks post challenge. Flow cytometric analysis was employed to evaluate frequencies of CD4+ and CD8+ lymphocytes with proinflammatory phenotypes: **(A, C)** IFNg producing T helper cells, **(B, D)** IFNg producing cytotoxic T cells. The difference among cohorts were assessed by two sample t-test. (*: P £ 0.05, **: P £ 0.01, ***: P £ 0.001, ****: P £ 0.0001)

**Figure 6:**
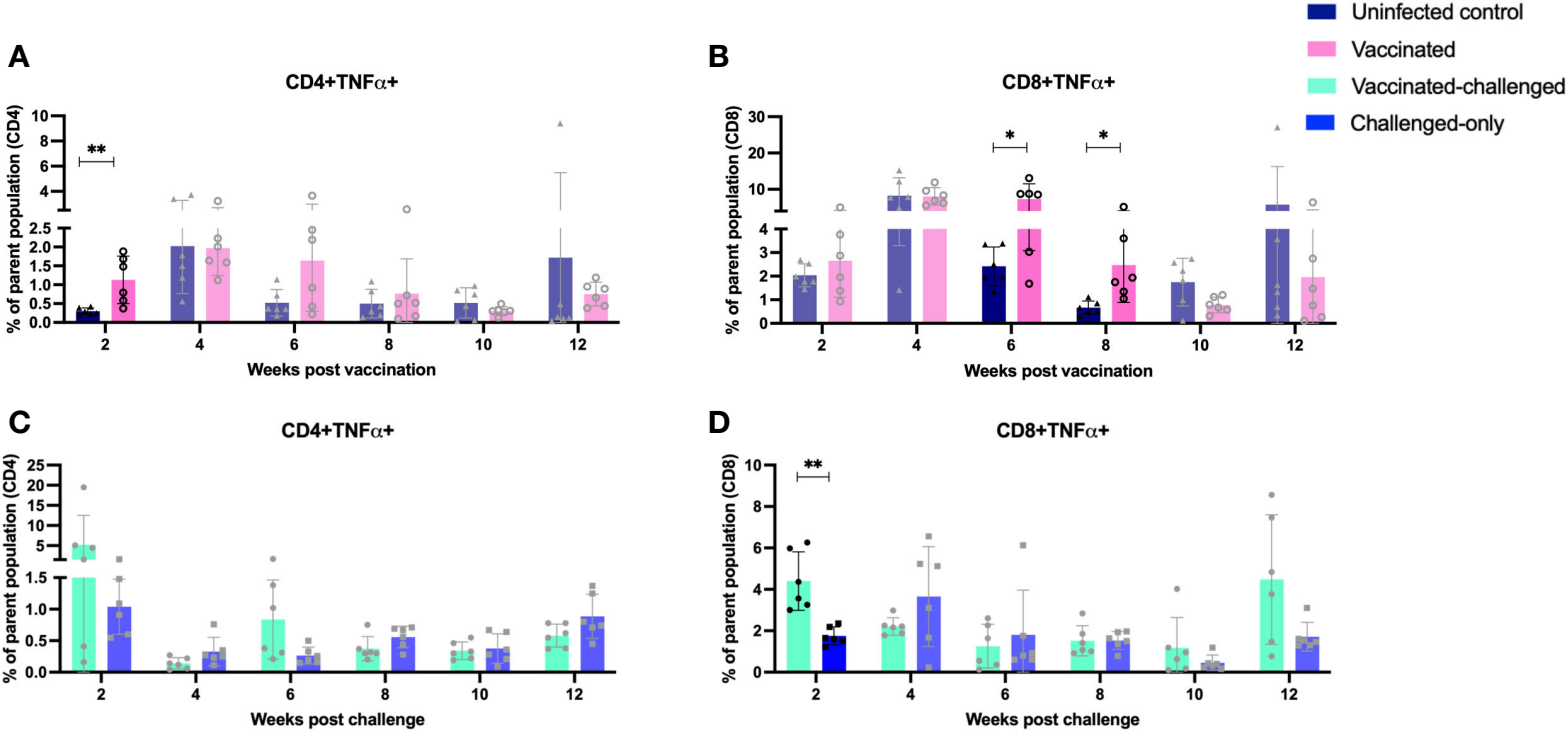
Immunophenotyping of TNFa-producing T cells. PBMCs were isolated from vaccinated and uninfected control cohorts at 2, 4, 6, 8, 10, 12 weeks post vaccination. PBMCs were isolated from vaccinated-challenged and challenged-only cohorts at 2, 4, 6, 8, 10, 12 weeks post challenge. Flow cytometric analysis was employed to evaluate frequency of CD4+ and CD8+ lymphocytes with proinflammatory phenotypes: **(A, C)** TNFa producing T helper cells, **(B, D)** TNFa producing cytotoxic T cells. The difference among cohorts were assessed by two sample t-test. (*: P £ 0.05, **: P £ 0.01, ***: P £ 0.001, P £ 0.0001).

The vaccinated cohort was directly compared to the uninfected control cohort, as their timepoints and ages aligned. This comparison allowed for evaluating the effects of vaccination on the induction of T cell responses. The vaccinated cohort had a significantly greater frequency of CD4+IFNγ+ cells at 8- and 10-weeks post-inoculation (**Figure 5A**) when compared to the uninfected control cohort. Further, the vaccinated cohort had a significantly greater frequency of CD8+IFNγ+ cells relative to the uninfected control cohort at 4-, 8-, and 12-weeks post-inoculation (**Figure 5B**). Moreover, the vaccinated cohort also had a significantly greater abundance of CD4+TNFα+ cells y at 2 weeks post-inoculation (**Figure 6A**). and CD8+TNFα+ cells at 6- and 8-weeks post-inoculation when compared to the uninfected control (**Figure 6B**).

The vaccinated-challenged cohort and the challenged-only cohort were compared to investigate the effect of vaccination on immune responses against enteric MAP infection. The vaccinated-challenged cohort had a significantly greater frequency of CD4+IFNγ+ cells compared to the challenged-only cohort at 8- and 10-weeks post-inoculation (**Figure 5C**). Additionally, the vaccinated-challenged cohort also had a significantly greater frequency of CD8+IFNγ+ cells compared to the challenged-only cohort at 8-, 10-, and 12-weeks post-inoculation (**Figure 5D**. Furthermore, at 2 weeks post-inoculation, the vaccinated-challenged cohort demonstrated a significantly higher frequency of CD8+TNFα+ cells compared to the challenged-only cohort (**Figure 6D**). We standardized all variables under our control; however, there remains variation in the frequency of CD4+TNFα+ T cells in the unvaccinated control cohort. This variation may stem from factors that could also impact the observed outcomes in the vaccinated animals. It is crucial to emphasize that the experimental conditions were consistent across all groups.

### 3.4 Temporal changes in peripheral blood regulatory T cells following oral *BacA* vaccination and MAP infection

The balance between proinflammatory and regulatory mechanisms is critical for an optimal immune response. Tregs play a role in controlling excessive inflammation and preventing immunopathology. Chronic intracellular infections are capable of inducing proliferation of cells with anti-inflammatory and regulatory phenotypes. To assess the frequency of induced regulatory T cells, flow cytometry analysis of PBMCs targeted the expression of CD25 (activation marker expressed on the surface of regulatory T cells) and FOXP3 (intracellular transcription factor associated with regulatory T cell function). Peripheral blood mononuclear cells (PBMCs) were isolated at 2, 4, 6, 8, 10, and 12 weeks after inoculation of the oral vaccine for the vaccinated cohort and after challenge with virulent MAP in vaccinated-challenged and challenged-only cohort.

**Figure 7:**
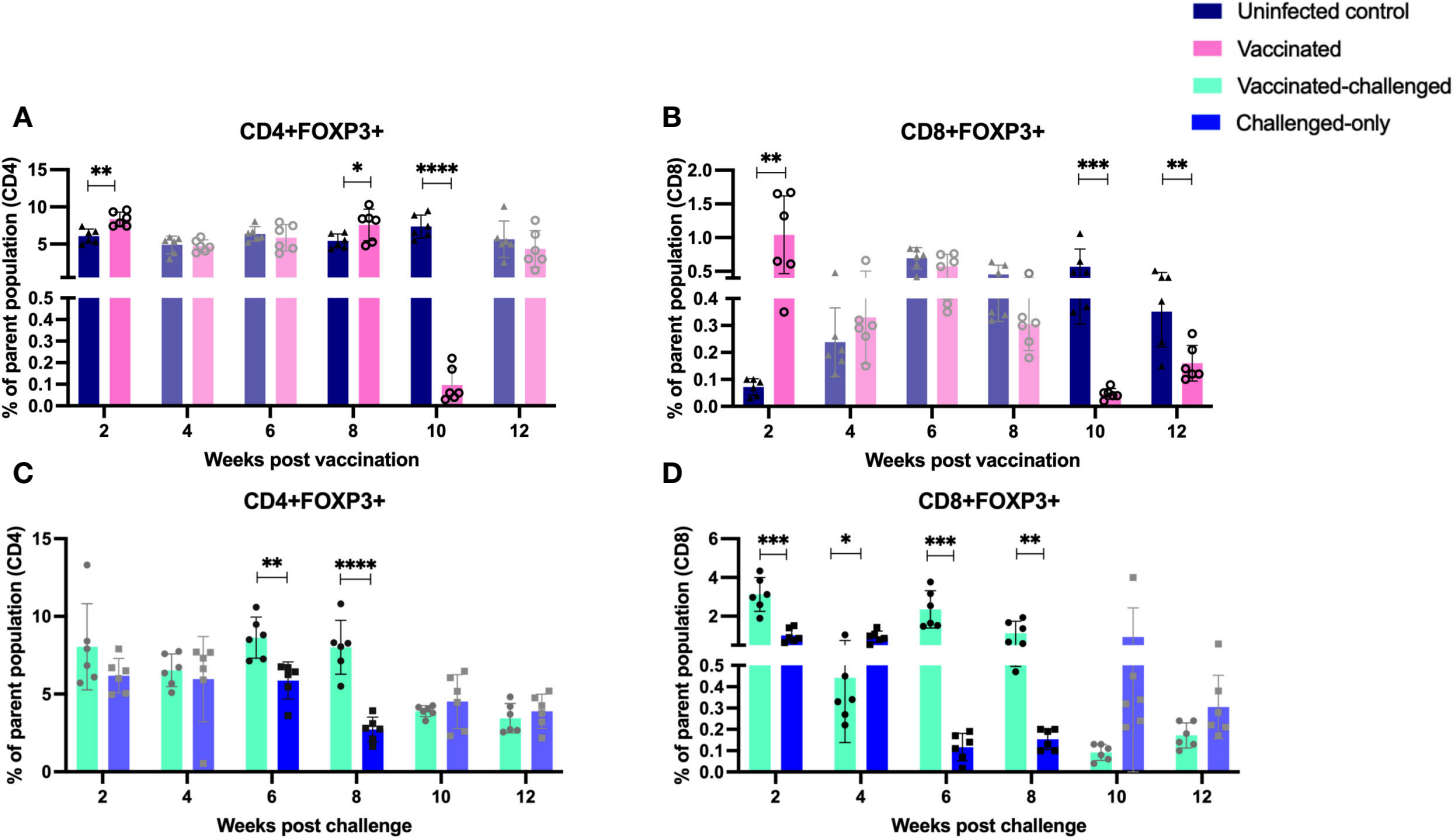
Immunophenotyping of T regulatory cells. PBMCs were isolated from vaccinated-challenged and challenged-only cohorts at 2, 4, 6-, 8-, 10-, and 12-weeks post-challenge, and from vaccinated and uninfected control cohorts at 2, 4, 6-, 8-, 10-, and 12-weeks post-vaccination. Flow cytometric analysis was used to measure the frequency of CD4+ and CD8+ T regulatory lymphocytes: **(A, C)** regulatory T helper cells, **(B, D)** regulatory cytotoxic T cells. The differences between cohorts were evaluated using a two-sample t-test. (*: P ≤ 0.05, **: P ≤ 0.01, ***: P ≤ 0.001, P ≤ 0.0001).

The vaccinated cohort was directly compared to the uninfected control cohort, as their timepoints and ages aligned. The vaccinated cohort showed a significantly greater frequency of induced regulatory T cells (CD4/CD8+CD25+FOXP3+) compared to the uninfected control cohort at 2- and 8-weeks post-inoculation. However, the frequency of induced regulatory T cells in the vaccinated cohort was significantly lower than that in the uninfected cohort at 10- and 12-weeks post-inoculation (**Figures 7A, B**).

The vaccinated-challenged cohort and the challenged-only cohort were compared to investigate the effect of vaccination on immune responses against enteric MAP infection. The vaccinated-challenged cohort exhibited a significantly greater frequency of induced regulatory T cells compared to the challenged-only cohort at 2-, 6-, and 8-weeks post-inoculation. However, at 4 weeks post-inoculation, the challenged-only cohort had a significantly greater frequency of CD8 regulatory T cells compared to the vaccinated-challenged cohort (**Figures 7C, D**).

### 3.5 Presence of peripheral T-cells with memory and activation phenotypes in vaccinated cohorts

**Figure 8:**
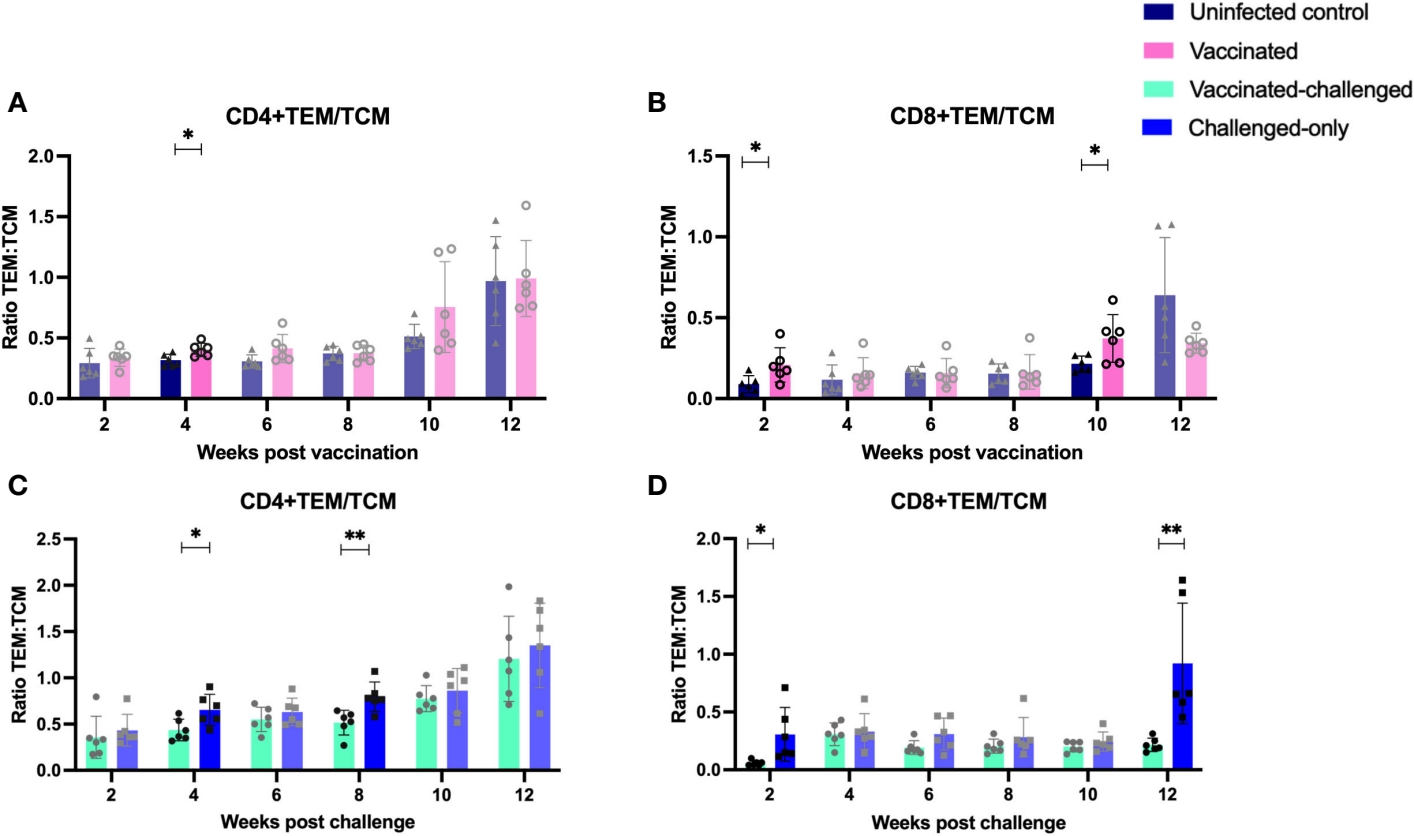
Ratio of effector memory to central memory CD4+ **(A, C)** and CD8+ **(B, D)** T lymphocytes. PBMCs were isolated from vaccinated-challenged and challenged-only cohorts at 2, 4, 6-, 8-, 10-, and 12-weeks post-challenge, as well as from vaccinated and uninfected control cohorts at 2, 4, 6-, 8-, 10-, and 12-weeks post-vaccination. The differences among cohorts were assessed using a two-sample t-test. (*: P ≤ 0.05, **: P ≤ 0.01, ***: P ≤ 0.001, P ≤ 0.0001).

The ratio of T effector memory cells (TEM) to T central memory cells (TCM) is a key factor in vaccination, as an optimal balance ensures a robust and sustained immune response. Effector memory cells contribute to the immediate defense against pathogens, while central memory cells provide long-term immunity by forming a reservoir of memory cells that can rapidly differentiate into effectors upon re-exposure to the same antigen, enhancing the vaccine’s overall efficacy. To assess the immediate memory response to MAP, the ratio of TEM/TCM) was calculated in all cohorts. At 4 weeks post-inoculation, the vaccinated cohort displayed a significantly higher TEM/TCM ratio of CD4+ cells compared to the uninfected control cohort. Similarly, at 2- and 10-weeks post-inoculation, the vaccinated cohort exhibited a significantly higher TEM/TCM ratio of CD8+ cells compared to the uninfected control cohort (**Figures 8A, B**).

The vaccinated-challenged cohort demonstrated a significantly higher TEM/TCM ratio of CD4+ TEM/TCM cells compared to the challenged-only cohort at 4- and 8-weeks post-inoculation. Similarly, the vaccinated-challenged cohort showed a significantly higher TEM/TCM ratio of CD8+ TEM/TCM cells compared to the challenged-only cohort at 2- and 12-weeks post-inoculation (**Figures 8C, D**).

**Figure 9:**
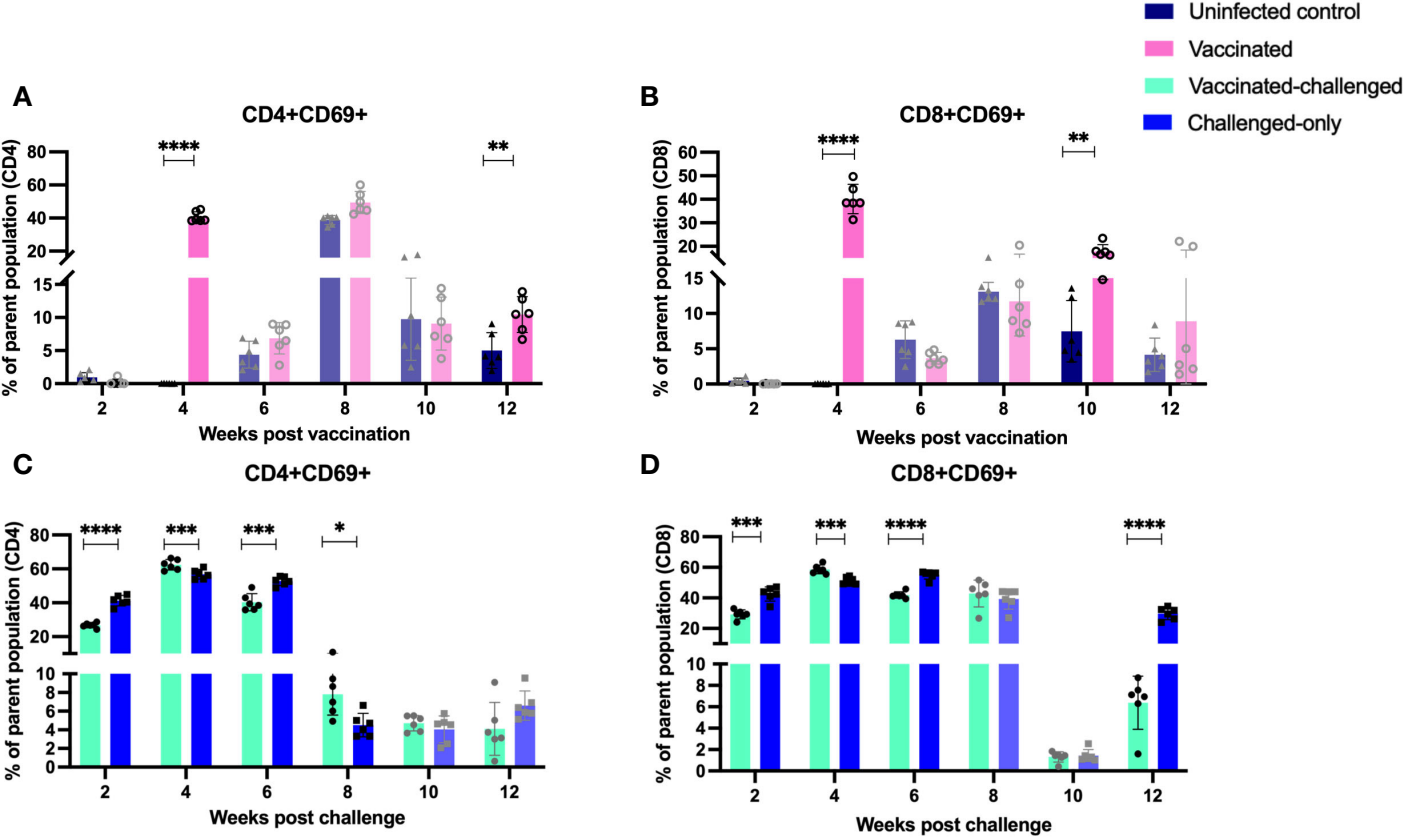
Immune cell profiling of PBMCs with activation marker. PBMCs were isolated from vaccinated-challenged and challenged-only cohorts at 2, 4, 6, 8, 10, 12 weeks post challenge and for vaccinated and uninfected control at 2, 4, 6, 8, 10, 12 weeks post vaccination. Flow cytometric analysis was employed to evaluate frequency of CD4+ **(A, C)** and CD8+ **(B, D)** lymphocytes with activation marker (CD69). The difference among cohorts were assessed by two sample t-test. (*: P £ 0.05, **: P £ 0.01, ***: P £ 0.001, P £ 0.0001).

Evaluation of the expression of early T cell activation marker (CD69) indicated that there is a variation in the kinetics of T cell activation between cohorts. At 4- and 12-weeks post-inoculation, the vaccinated cohort exhibited a significantly higher frequency of CD4+ and CD8+ T cells expressing the early activation marker compared to the uninfected control cohort (**Figures 9A, B**). Similarly, at 4- and 8- weeks post inoculation the frequency of CD4+ and CD8+ T cells with early activation marker was significantly greater in vaccinated-challenged cohort compared to challenged-only (**Figures 9C, D**).

### 3.6 Stimulation of PBMCs with MAP whole cell lysate induces expression of pro-inflammatory cytokines in vaccinated-challenged cohort

To ensure a specific immune response to MAP and assess the functionality of immune cells, their gene expression was measured following *in vitro* exposure to MAP antigens. Real-time RT-qPCR was used to quantify changes in cytokine gene expression for *IFN-γ*, *TNF-α*, *CXCL-10*, *IL-12*, *IL-18*, *IL-27*, *IL-2*, *IL-15*, and *IL-17*in PBMCs re-stimulated with MAP whole cell lysate. Cytokine gene expression was compared between the vaccinated-challenged cohort, the challenge-only cohort at 10 and 12 weeks after the challenge, and between the uninfected control and vaccinated cohorts at 10 and 12 weeks after vaccination.

**Figure 10:**
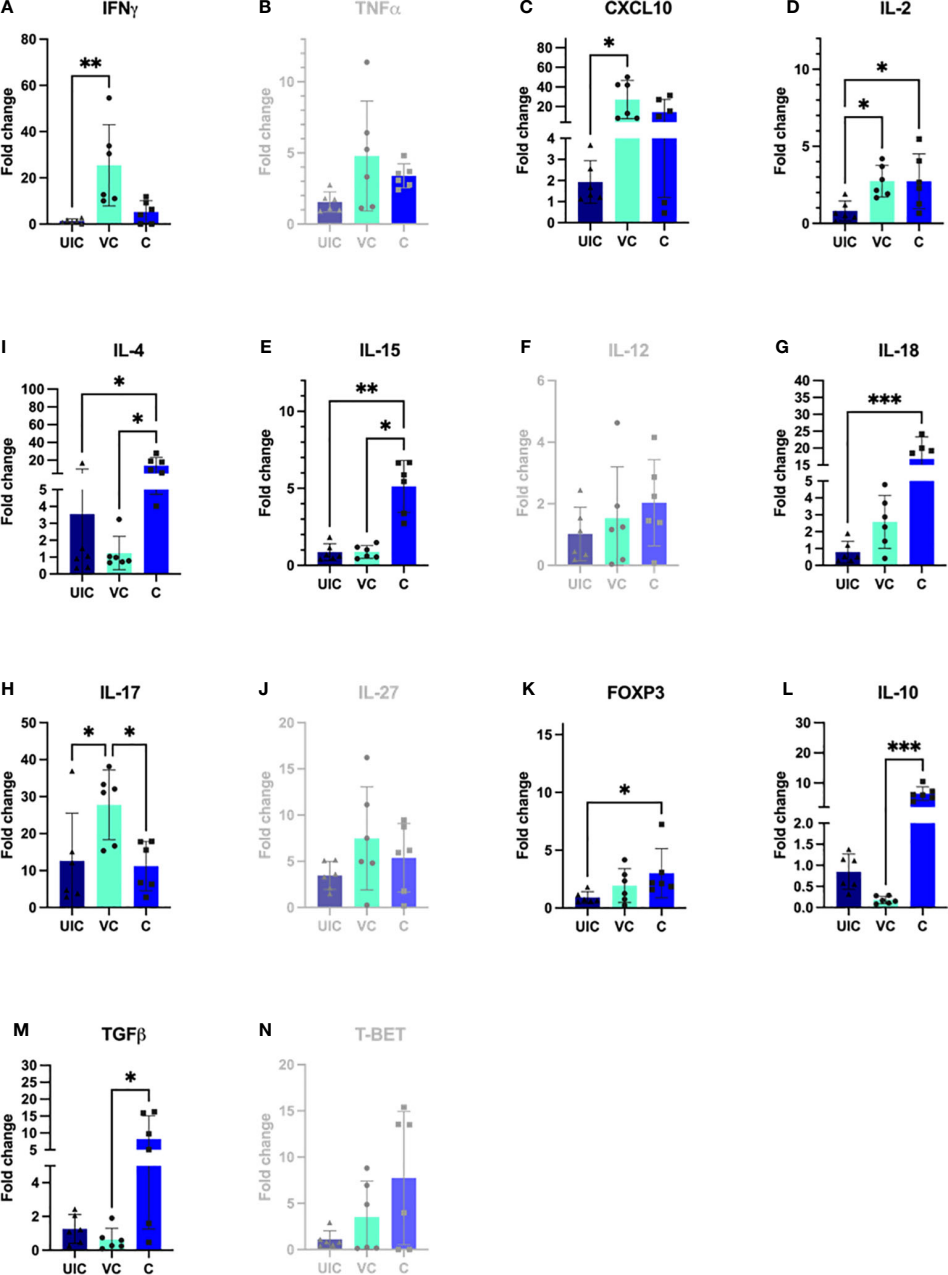
Antigen-specific cytokine responses in peripheral blood leukocytes. PBMCs were isolated from uninfected control, vaccinated-challenged, and challenged-only cohort at 10 weeks post challenge (4 weeks before necropsies) and re-stimulated in vitro with MAP whole cell lysate for 24 h. Cytokine gene expression was quantified in re-stimulated and resting cells using real-time RT-qPCR and normalized to H3F3A. Relative gene expression was calculated using 2-^ΔΔct^ and the difference among cohorts were assessed by one-way ANOVA and then further validated by Tukey-Kramer test. (UIC, uninfected control; VC, vaccinated-challenged; C, challenged-only) (*: P ≤ 0.05, **: P ≤ 0.01, ***: P ≤ 0.001, P ≤ 0.0001).

*IL-2* gene expression was significantly higher in both the vaccinated-challenged and challenged cohorts compared to uninfected control at 10 weeks post vaccination (**Figure 10D**). At 10 weeks post-challenge, the vaccinated-challenged cohort showed significantly greater transcript abundance for *IFN-γ*, and *IP-10* compared to the uninfected control cohort (**Figures 10A, C**). Additionally, the vaccinated-challenged cohort showed significantly greater *IL-17* expression compared to both the uninfected control and the challenged-only cohorts (**Figure 10H**).

At 10 weeks post-inoculation, the challenged-only cohort showed significantly greater *IL-18* transcript abundance when compared to the uninfected control cohort (**Figure 10G**), and significantly higher *IL-15* transcript abundance compared to both vaccinated-challenged and uninfected control cohorts (**Figure 10E**).

**Figure 11:**
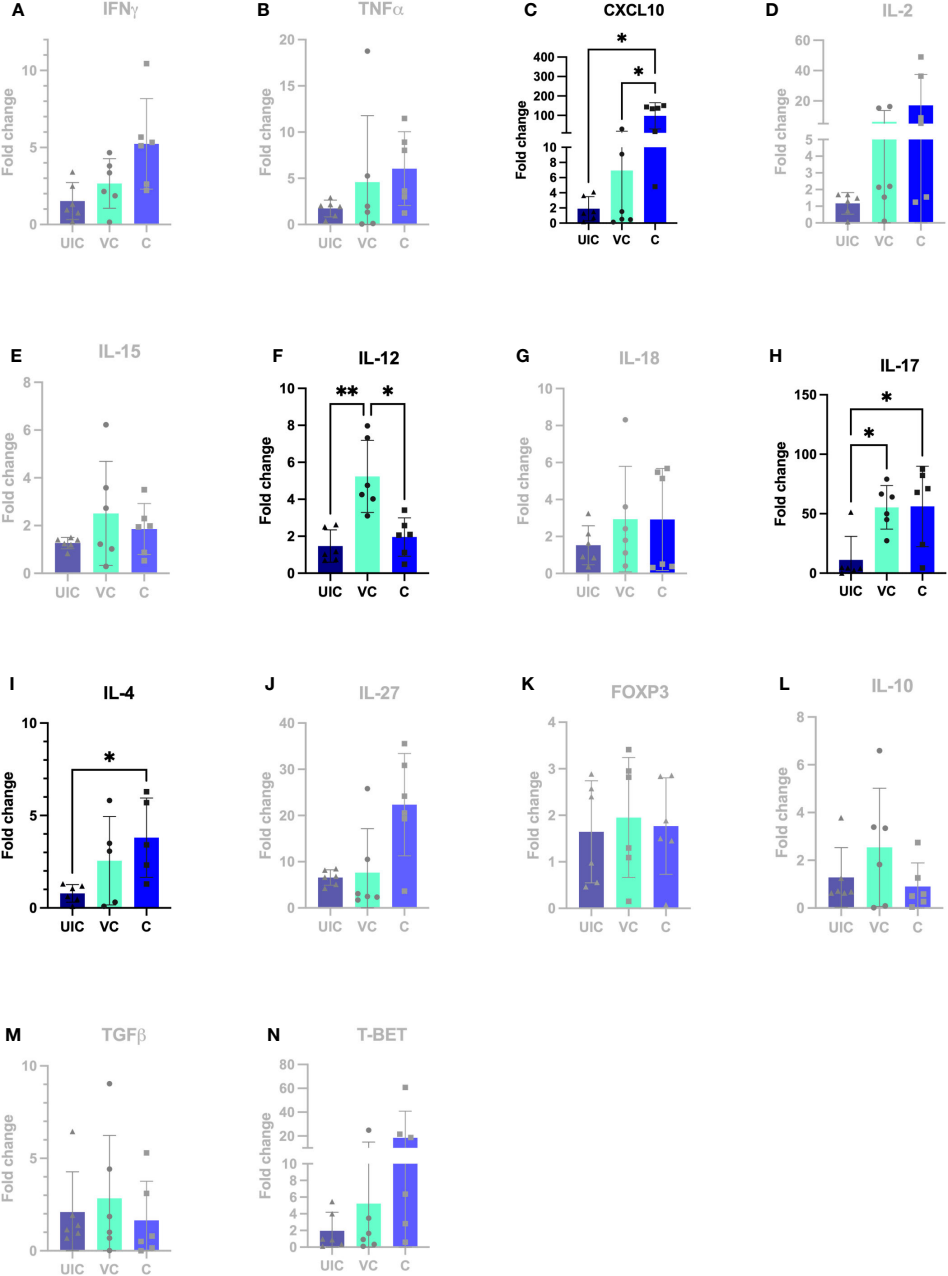
Antigen-specific cytokine responses in peripheral blood leukocytes. PBMCs were isolated from uninfected control, vaccinated-challenged, and challenged-only cohort at 12 weeks post challenge (2 weeks before necropsies and re-stimulated in vitro with MAP whole cell lysate for 24). Cytokine gene expression was quantified in re-stimulated and resting cells using real-time RT-qPCR and normalized to H3F3A. Relative gene expression was calculated using 2-^ΔΔct^ and the difference among cohorts were assessed by one-way ANOVA and then further validated by Tukey-Kramer test. (UIC, uninfected control; VC, vaccinated-challenged; C, challenged-only) (*: P ≤ 0.05, **: P ≤ 0.01).

At 12 weeks post-inoculation, both vaccinated-challenged and challenged-only cohorts had significantly higher *IL-17* expression compared to uninfected cohort (**Figure 11H**). Additionally, the vaccinated-challenged cohort indicated significantly higher expression levels of *IL-12* compared to challenged-only and uninfected control cohorts (**Figure 11F**).

At 12 weeks post-inoculation, the challenged-only cohort had significantly higher *IP-10* expression compared to vaccinated-challenged and uninfected control cohorts (**Figure 11C**).

No significant differences in gene expression were identified between vaccinated and vaccinated-challenged cohort. Only at 12 weeks post vaccination, the vaccinated-challenged cohort has significantly higher *IL-12* gene expression compared to vaccinated and uninfected control cohorts.

**Figure 12:**
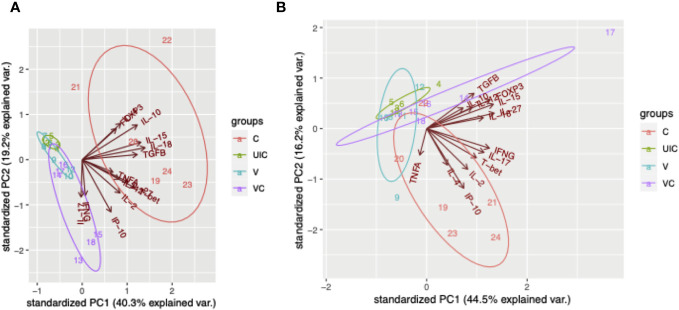
Principal component analysis. **(A)** PCA for gene expression at 10 weeks post inoculation. **(B)** PCA for gene expression at 12 weeks post inoculation. Cohorts are color coded, and numbers indicate animal IDs. Each cohort is clustered with a different ellipse. The vectors of the loading plots showing how strongly each variable influence a principal component, with their directions indicating how different variables are correlated with one another. (UIC, uninfected control; V, vaccinated; VC, vaccinated-challenged; C, challenged-only).

According to the principal component analysis (PCA), there is a positive correlation between the observations of the challenged-only cohort and the variables *IL-4*, *TNFα*, and *IL-2* at both 10- and 12-weeks post inoculation, as shown in **Figures 12A, B**. Furthermore, the observations of the challenged-only cohort have positive correlation with anti-inflammatory cytokines including *FOXP3*, *IL-10*, and *TGFβ* at 10 weeks post inoculation. However, the pattern of observations in the vaccinated-challenged cohort can be better explained by the variables *IL-17* and *IFN-γ* at 10 weeks post inoculation. Both vaccinated cohorts cluster closer together at both time points. In contrast, the challenged-only cohort exhibits a larger variation, and the cluster of observations is more spread out. The clear deviation of the challenged-only cohort’s direction in the biplot implies that the variables that are critical for distinguishing this cohort show divergent patterns or relationships compared to the other cohorts. Specifically, this difference can be observed in the directions of some significant cytokines, including *IL-4*, *TNFα*, *IL27*, *IL-10*, *FOXP3*, *IL-15*, *IL-18*, and T-bet at 10 weeks post inoculation, as well as *IL-4*, *IL-2*, *TNFα*, and *IP-10* at 12 weeks post inoculation (**Figure 12**).

### 3.7 The expression of anti-inflammatory cytokines is highly upregulated in challenged-only cohort

The study aimed to investigate a panel of anti-inflammatory cytokines including *IL-4*, *TGFβ*, and *IL-10*, and two transcription factors including T-bet and *FOXP3*. At 10 weeks post-challenge the expression of *IL-10* and *TGFβ* were downregulated in vaccinated-challenged cohort. However, the expression levels of *IL-10* and *TGFβ* in challenged-only cohort were upregulated and significantly higher than vaccinated-challenged cohort (**Figures 10L, M**). Additionally, the challenged-only cohort had a higher expression of *IL-4* compared to vaccinated-challenged and uninfected control cohorts (**Figure 10I**). The expression of transcription factor *FOXP3* was also higher in challenged-only cohort compared to uninfected control at 10 weeks post-challenge (**Figure 10K**).

At 12 weeks post-challenge, the expression levels of *IL-4* anti-inflammatory cytokine was higher in challenged-only cohort compared to uninfected control cohort (**Figure 11I**).

## 4 Discussion

The present study was designed to assess the effectiveness of the oral *BacA* vaccine in preventing MAP infection in dairy calves and to investigate the immune responses elicited by oral vaccination against MAP. The findings of the present research suggest that the live attenuated oral *BacA* vaccine offers a degree of protection by decreasing intestinal MAP burden in the ileum and ileocecal valve. However, the vaccine did not reduce MAP infection in the jejunum, jejunal MLN, ileal MLN, and ileocecal valve. The oral *BacA* vaccine did demonstrate efficacy in reducing MAP shedding in feces starting at eight weeks post-challenge. Oral *BacA* vaccine efficacy was associated with the induction of pro-inflammatory immune responses evidenced by the increased abundance of IFN-γ and TNF-α producing CD4+ T helper cells and CD8+ cytotoxic T cells. This study represents the first evaluation of an oral modified live MAP vaccine against an oral challenge in young calves. The benefits of an oral MAP vaccine include imitating the natural infection route, convenient, non-invasive administration of the vaccine, induction of local immunity at the site of infection, and potential benefits to herd-level protection by reducing shedding and transmission. It is also important to consider the current limitations including variable efficacy due to seeding of the modified live MAP might not be uniform throughout the gastrointestinal tract, and limited control over individual dosing and bacterial load due to possible re-exposure and reinfection (29).

The oral *BacA* vaccine strain was undetectable in any of the intestinal tissues sampled from the vaccinated calves. This is consistent with our previous observations (19), where the *BacA* vaccine strain could not be detected in intestinal tissue four months post-inoculation yet still retained its capacity to induce host immune responses. This study aimed to evaluate the effectiveness of the oral *BacA* vaccine in preventing enteric MAP infection. Our results demonstrated that oral vaccination with *BacA* was most effective at controlling MAP infection, to undetectable levels, in the continuous Peyer’s patches located in the terminal small intestine and also the Peyer’s patch associated with the ileocecal valve. However, there were no significant differences observed in MAP bacterial burden in the discrete Peyer’s patches of the jejunum or MLNs draining each targeted intestinal site. Due to the absence of tissue samples collected at early timepoints, we cannot draw any conclusions regarding which tissue sites were or were not colonized by MAP in the early stages after the challenge. The investigation of an alternate MAP variant, like the *relA* mutant, has shown that introducing a *relA* vaccine via an ileal canulation model results in a partial reduction of bacterial load in the tissues (30). In young calves, the continuous Peyer’s patches, which occupies the terminal small intestine and continues into the ileocecal fold is a major portal of entry for MAP (26, 31–33). Therefore, an effective vaccine for cattle needs to elicit local immune responses at this intestinal site to protect against persistent MAP infection. Histological analyses revealed only two animals from the vaccinated-challenged cohort that had focal lesions in small intestine in contrast to challenged-only cohort which had five animals with focal and multifocal lesions. As the delivery of the modified live *BacA* vaccine mimics the natural route of MAP exposure (34, 35), our data suggests that enteric vaccination can induce host immune responses that offer protection to the terminal small intestine (specifically the continuous Peyer’s patch) but not to the discrete Peyer’s patches in the jejunum. It has been previously demonstrated that MAP induces differential immune responses in continuous and discrete Peyer’s patches (31, 34). Thus, it is likely that the oral *BacA* vaccine induces differential local immunity depending on the intestinal site it colonizes. Given the functional differences between the discrete and continuous Peyer’s patches, further studies are warranted to investigate the capacity of oral or enteric vaccines to induce local immunity at these distinct anatomical sites.

The feces culture and qPCR indicate that MAP shedding was happening in both vaccinated and non-vaccinated animals during the early stage of infection. At two, four- and six-weeks post-inoculation, several animals were positive for MAP in their fecal cultures from all inoculated cohorts. From eight weeks post-inoculation onwards, MAP was undetectable in calves belonging to the vaccinated and vaccinated-challenged cohorts. However, in the challenged-only cohort, one animal continued to test positive for MAP during those time points. It is important to mention that the detection of MAP at younger ages has its own limitations related to intermittent shedding with lower bacterial loads and decrease in sensitivity due to decontamination steps before culturing.

In our prior trial, we observed notable differences between *BacA* vaccine and wildtype MAP control groups regarding CD4+CD45RO+ cell frequency and the expression of proinflammatory genes, including IFNγ (19). Both groups were inoculated at 2 weeks of age. In the current study, vaccinated-challenge and challenge-only groups were introduced to explore the impact of vaccination on immune responses to wildtype MAP. This investigation aims to determine if administering a live-attenuated strain three weeks before challenge enhances cell-mediated immune responses and mitigates immune system suppressions caused by wildtype MAP. In this scenario, the reference point is the time post-challenge. Flow cytometric analysis of peripheral blood mononuclear cells (PBMCs) at various time points post inoculation revealed that the oral *BacA* vaccine induces the pro-inflammatory immune response during the early stages of infection, as well as later timepoints. Furthermore, comparing immune responses in the *BacA* vaccinated cohort to the uninfected cohort provided a basis for interpreting vaccine-induced immune responses in the vaccinated-challenged cohort compared to the challenged-only cohort. The analysis of CD4 and CD8 cells producing IFNγ and TNFα revealed that the frequency of immune cells with a proinflammatory phenotype is significantly greater in the vaccinated cohorts compared to the uninfected and challenge-only cohorts. A significantly greater frequency of CD4+IFNγ+ cells at four-, eight- and ten-weeks post-inoculation, as well as a significantly greater frequency of CD8+IFNγ+ cells at eight- and twelve-weeks post-inoculation, was observed in both the vaccinated and vaccinated-challenged cohorts compared to the uninfected control and challenged-only cohort, respectively. These findings provide evidence for the potential correlates of protection induced by the oral *BacA* vaccine. Furthermore, a significantly greater frequency of CD8+TNFα+ cells at two weeks post-inoculation was observed in the vaccinated-challenged cohort compared to the challenged-only cohort. Additionally, a significantly greater frequency of CD4+TNFα+ cells at two weeks post-inoculation and CD8+TNFα+ cells at six- and eight-weeks post-inoculation was observed in the vaccine cohort compared to the uninfected cohort. These results indicate the dominance of proinflammatory immune cells in the vaccinated cohorts that initiate as early as two weeks post-inoculation and continue to persist to later time points but also. The increased frequency of these proinflammatory cells suggests that oral *BacA* vaccination can potentially afford both early protection and provide durable immunity out to 12 weeks post-challenge. Long-lasting immunity is critical for a JD vaccine given the persistent nature of MAP infections and the likelihood of exposure and re-exposure throughout the lifetime of the animal. It is important to note that the mere presence of T helper cells cannot be regarded as a sufficient correlate of protection. Immediate effector functions of memory cells are essential upon re-exposure to intracellular pathogens (35).

In this study, the ratio of CD4 TEM/TCM was higher four weeks after inoculation, while the ratio of CD8 TEM/TCM was higher at two- and ten-weeks post inoculation in the vaccinated cohort compared to the uninfected control cohort. However, the ratios of CD4 TEM/TCM and CD8 TEM/TCM were never higher in the vaccinated-challenged cohort compared to the challenged-only cohort. The assessment of the ratio between T effector memory cells (TEM) and T central memory cells (TCM) enabled the evaluation of memory response and the preparedness of each treatment cohort to respond to reinfection. TEM cells are memory cells that are poised to perform effector functions immediately upon reencountering the antigen. They primarily reside in peripheral tissues and exhibit rapid production of effector cytokines and cytotoxic activity. On the other hand, TCM cells have the ability to migrate to secondary lymphoid organs and sustain the memory response by generating new TEM cells (36). The ratio of TEM to TCM cells can vary depending on the stage of infection and the individual’s immune status. During an active mycobacterial infection, there is usually an increase in the proportion of TEM cells, reflecting the ongoing immune response and the need for effector functions to control the infection (37, 38). This is often accompanied by a decrease in the proportion of TCM cells. The shift towards TEM cells suggests a more immediate and robust immune response. Conversely, in the context of vaccine-induced immune responses or during the resolution of the infection, there may be a shift towards a higher proportion of TCM cells (39). This indicates the establishment of long-term memory and a transition to a more quiescent immune state (40). The findings are consistent with the observation of lower infection burdens in the vaccinated-challenged cohort compared to the challenged-only cohort, suggesting a dominance of TCM cells in the former. These results align with the assessment of CD4+CD69+ cells, which are activated T cells that rapidly express CD69 upon immune cell activation. The consistent presence of CD4+CD69+ cells in peripheral blood indicates the chronic presence of antigen and activation of T cells (41). These activated T cells were observed only at earlier time points (four- and eight-weeks post inoculation) in the vaccinated cohorts. There is also evidence of T cells specific to *Mycobacterium tuberculosis* being first detected 2-3 weeks after infection (42). Johne’s disease is prevalent in young newborn animals, especially on positive farms, where immediate exposure to MAP occurs through contaminated food and the environment. To mimic this natural scenario, a calf challenge trial was conducted, assessing immune cell composition at early and late timepoints (2 to 12 weeks) post-vaccination/challenge.

The presence of a significantly greater abundance of Treg cells in the vaccinated and vaccinated-challenged cohorts at two, six-, and eight-weeks post-inoculation may be a result of excessive proinflammatory responses during those early time points. This is supported by the observation of a great frequency of CD4+TNFα+ cells at the same time points. It is important to note that the role of Tregs in mycobacterial infections is complex and context dependent. While Tregs contribute to maintaining immune homeostasis and preventing immunopathology by balancing immune responses and immune regulation without impacting the maintenance of effector cells, they also have the ability to suppress the activation and effector functions of other immune cells (43). T cells disrupt intestinal homeostasis by inducing damage through the destructive effects of IFNγ on Paneth cells (44). This process may trigger anti-inflammatory responses aimed at preventing host damage. This regulatory function of Tregs helps in maintaining immune balance and preventing excessive inflammation. However, it can also have implications for the efficacy of vaccines (45, 46). There is evidence indicating the expansion of regulatory T cells (Treg cells) and the induction of epigenetic modulation in Treg cells following vaccination with the BCG live attenuated vaccine (47, 48). Further research is needed to understand the precise mechanisms and optimal balance between Tregs and other immune cells for effective control of mycobacterial infections and vaccine-induced immunity.

The analysis of the cytokine profile in stimulated peripheral blood mononuclear cells (PBMCs) indicated that the increased frequency of CD8+IFNγ+ cells observed at 10 weeks post-inoculation is consistent with the high expression of *IFNγ* and *IP-10* at the same time point. Although both the vaccinated-challenged and challenged-only cohorts showed a combination of pro- and anti-inflammatory responses, the challenged-only cohort exhibited higher expression of anti-inflammatory cytokines, including *IL-4*, *IL-10*, and *TGFβ*, compared to the vaccinated-challenged cohort. This finding is in line with the higher expression of the transcription factor *FOXP3*, associated with regulatory T cells (Tregs), in the challenged-only cohort compared to the uninfected cohort. However, *IL-10* and *TGFβ* were downregulated in the vaccinated cohorts. Considering the expectation that a vaccine would facilitate a faster immune response, it is evident that the immune responses observed at 10 weeks post-inoculation in the vaccinated cohorts are occurring at a later time point, specifically 12 weeks post challenge, in the challenged-only cohort. Also, the immune cells trafficking and accumulating in blood were perhaps generated much earlier at the local site of vaccination/infection. Principal Component Analysis (PCA) also suggests a significant variation in cytokine expression among animals in the challenged-only cohort. Conversely, the cytokine expression in the vaccinated cohorts is more homogeneous and closely similar to each other. The clear deviation of the challenged-only cohort’s direction in the biplot implies that the variables that are critical for distinguishing this cohort show divergent patterns or relationships compared to the other cohorts. The pattern of cytokine expression of the challenge-only cohort is explained by anti-inflammatory variables at earlier timepoint. Overall, the vaccinated cohorts exhibit a consistent and synchronized cytokine expression, while the challenged-only cohort shows a delayed cytokine expression with wider variation. Transient infection with *BacA* induces a memory response capable of mounting a rapid reaction to subsequent MAP infection. Gene expression is influenced by age, gender, genetics, and environmental factors. To control for these variables, we maintained uniform age and environmental conditions across all groups. This standardization enabled the observation of specific responses to MAP in inoculated animals, providing a more accurate understanding of the studied gene expressions.

In summary, the study suggests that oral vaccination with oral *BacA* vaccine offers partial protection in the continuous Peyer’s patches of the ileum, which is a major site of MAP infection in young calves. The potential protective effect of the vaccine is associated with the presence of a higher frequency of T cells displaying pro-inflammatory phenotypes and indications of memory establishment. However, it is important to acknowledge the limitations of the study. These include the short duration of the trial, the evaluation of only one vaccine dose, only one challenge dose, only one timing for the vaccination and the challenge, the use of a high challenge dose to minimize the risk of underdosing, and the variations introduced by the oral infection model. The high challenge dose employed may underestimate the vaccine’s efficacy in a scenario of gradual exposure to a low dose of MAP under natural conditions. Moreover, as blood contains immune cells trafficking from different mucosal immune compartments, it cannot be determined where these cells are trafficking from or where the induction of these antigen-specific cells occurred. Despite these limitations, the promising results observed in this study warrant further investigation into mucosal immune responses in the intestine elicited by the vaccine. To optimize oral vaccination for ruminants, it is essential to take into account the variations in the small intestine across different regions to ensure the stimulation of mucosal immunity throughout the entire gastrointestinal tract. Further optimization and formulation of the vaccine is also needed to determine if the efficacy of the vaccine can be improved, evaluate the duration of immunity, and optimizing the vaccine with adjuvants induce earlier onset of immunity. In conclusion, while the study has limitations, it provides valuable insights into the potential of the oral *BacA* vaccine to protect dairy calves against MAP infection. Future studies can build upon these findings to gain a deeper understanding of mucosal immune responses and explore the use of the oral *BacA* vaccine in combination with other vaccines for enhanced protection in a calf infection trial and in natural infection under field conditions.

## Data availability statement

The raw data supporting the conclusions of this article will be made available by the authors, without undue reservation.

## Ethics statement

The animal study was approved by The calf study was reviewed and approved by the Veterinary Science Animal Care Committee of the University of Calgary. Animal care protocol AC21-0199 was implemented and all procedures were carried out in accordance with the Standard Operating Procedures (SOPs) specified therein. The study was conducted in accordance with the local legislation and institutional requirements.

## Author contributions

RE: Conceptualization, Data curation, Formal analysis, Investigation, Methodology, Visualization, Writing – original draft, Writing – review & editing. AF: Conceptualization, Investigation, Methodology, Writing – review & editing. VH-M: Investigation, Writing – review & editing. OI: Investigation, Writing – review & editing. JDB: Conceptualization, Funding acquisition, Investigation, Project administration, Resources, Supervision, Validation, Writing – review & editing.
